# Novel synthesis of siligraphene/tungstates (g-SiC/AWO) with promoted transportation of photogenerated charge carriers via direct Z-scheme heterojunctions

**DOI:** 10.1038/s41598-023-37170-5

**Published:** 2023-06-20

**Authors:** Maryam Afsharpour, Somayeh Darvishi-Farash

**Affiliations:** Department of Inorganic Chemistry, Chemistry and Chemical Engineering Research Center of Iran, Tehran, 14335-186 Iran

**Keywords:** Environmental chemistry, Inorganic chemistry, Chemical synthesis, Materials for energy and catalysis, Nanoscale materials

## Abstract

We developed here the efficient photocatalysts for the removal of high concentrations of tetracycline under visible light by immobilizing the AWO (A = Ag, Bi, Na) nanocrystals on the surface of siligraphene (g-SiC) nanosheets. The g-SiC/AWO composites was synthesized by magnesiothermic synthesis of g-SiC and sonochemical immobilization of tungstates. These new heterojunctions of g-SiC/tungstates show superior photocatalytic activities in the degradation of high concentrations of tetracycline and 97, 98, and 94% of tetracycline were removed by using low amounts of g-SiC/Ag_2_WO_4_, g-SiC/Bi_2_WO_6_, and g-SiC/Na_2_WO_4_ catalysts, respectively. Based on band structures, the band gaps reduce and the photocatalytic activities were extremely enhanced due to the shortening of electron transfer distance through the Z-scheme mechanism. Also, the graphenic structure of g-SiC is another parameter that was effective in improving photocatalytic performance by increasing the electron transfer and decreasing the rate of electron–hole recombination. Furthermore, the π back-bonding of g-SiC with metal atoms increases the electron–hole separation to enhance the photocatalytic activity. Interestingly, g-SiC composites (g-SiC/AWO) showed much higher photocatalytic properties compared to graphene composites (gr/AWO) and can remove the tetracycline even at dark by producing the oxygenated radicals via adsorption of oxygen on the positive charge of Si atoms in siligraphene structure.

## Introduction

Today, with the progress of human civilization and population growth, we are facing a severe water shortage and reduction of water quality due to various pollutions^1,2^. Wastewaters treatments are very expensive, and traditional methods in addition to imposing heavy costs do not meet the relevant standards in many cases. Recently, with the introduction of new technologies, new solutions such as novel photocatalysts, membranes, and environmentally friendly adsorbents have been introduced for wastewater treatments^3–6^. Photocatalysts are an important class of nanomaterials used in the purification of heavily polluted water and are very effective when other purification methods are not useful or economical^7–10^.

Among the wide range of metal-containing photocatalysts^7–17^, the tungstates with the general formula of A_2_WO_x_ (AWOs) are considered to be the promising candidates with great photocatalytic potentials due to the unique electronic structure, wide band gap, good chemical stability, and physicochemical properties^18–28^. However, the high recombination rate of photogenerated electron–hole pairs reduces the potential of these catalysts to remove the high concentration pollutants^18–28^. To overcome this limitation, these photocatalysts combined with other materials to reduce the recombination rate. Graphene and its derivations are wildly used as metal-free co-catalysts to enhance photocatalytic performance by promoting charge transfer and reducing the rate of electron–hole recombination^24–31^.

Also, interface engineering such as the fabrication of heterojunctions is an effective solution to improve the photogenerated charge separation in photocatalysts. In addition to 2D metal-containing semiconductors such as MXenes^32^, metal-free semiconductors such as g-C_3_N_4_ and g-SiC are also considered to be the most promising material for the construction of composite heterojunctions^7,33–39^.

Siligraphenes (g-SiCs) are the class of graphenic materials in which a large number of carbon atoms have been replaced by silicon^40–42^. The g-SiC has high thermal, mechanical, and chemical stability along with high surface area, low band gap, and high electron mobility which made it a superior catalyst for removing different organic pollutants at high concentrations^42–47^. The 2D layered structure of siligraphene made it superior candidate to form the well-defined composite heterojunctions. Siligraphene can prevent the aggregation of composites as well as improve the separation of photogenerated electron − hole pairs due to the superior electron mobility of siligraphene. Also, the doping of Si atoms in this structure gives extraordinary properties to this compound compared to graphene, which can make this material a very good candidate for making improved catalysts with graphenic structures. So far none has reported the potential of siligraphene coupled with tungstates (AWOs) for removal of organic pollutants in water.

In this work, the new Z-scheme heterojunctions of Ag_2_WO_4_, Bi_2_WO_6_, and Na_2_WO_4_ with siligraphene (g-SiC) were synthesized for removal of high concentration of tetracycline under visible light. Siligraphene (g-SiC) was synthesized via a low-temperature method to maintain the porous structure and g-SiC/AWO composites were developed by immobilizing of tungstates on the surface of g-SiC. Siligraphene can decrease the band gap, increase the charge transfer, and decrease the rate of photogenerated electron–hole recombination.

## Result and discussion

### Characterization of g-SiC/Ag_2_WO_4,_ g-SiC/Bi_2_WO_6_, and g-SiC/Na_2_WO_4_

**Figure 1:**
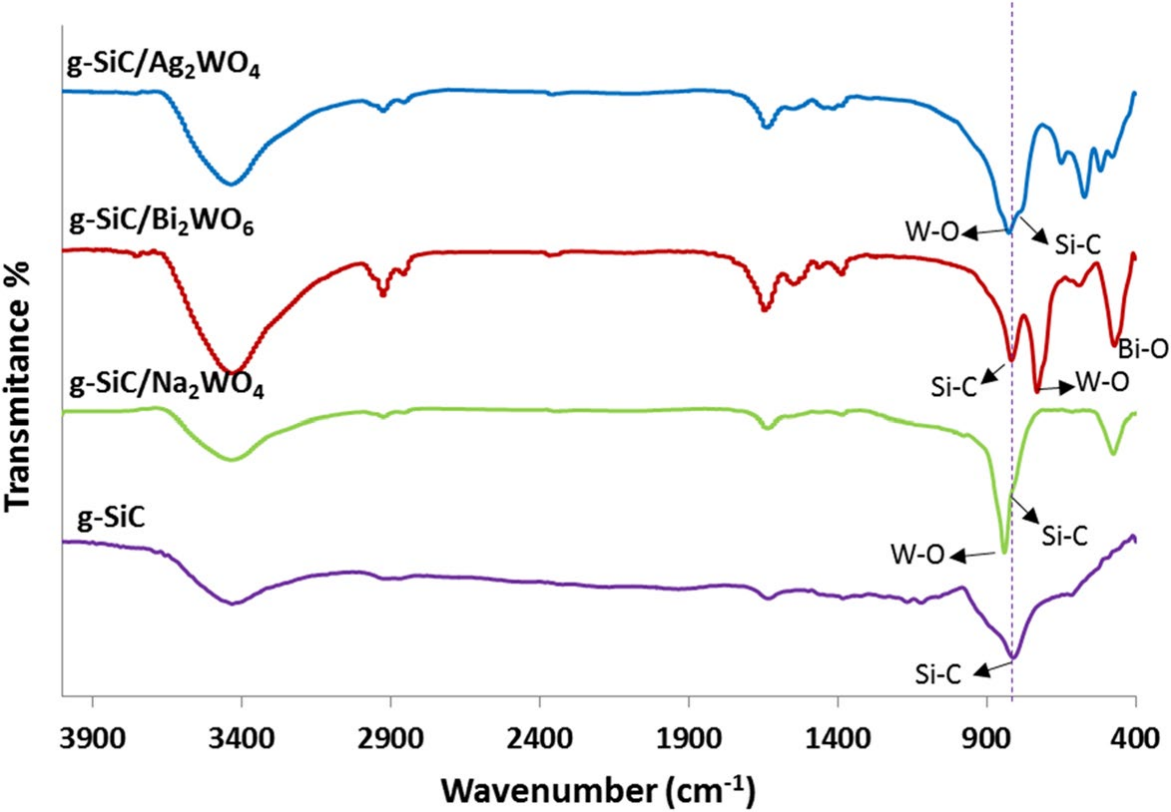
FT-IR spectra of g-SiC, g-SiC/Ag_2_WO_4,_ g-SiC/Bi_2_WO_6_, and g-SiC/Na_2_WO_4_.

Figure 1 shows the FT-IR spectra of g-SiC catalysts. FT-IR spectra of g-SiC shows the Si–C stretching vibration at 813 cm^-1^ which confirms the formation of a bond between silicon and carbon. The g-SiC/Ag_2_WO_4_ spectra shows the stretching vibration of the Si–C bond at 785 cm^-1^ which is shifted to a lower frequency compared to g-SiC (813 cm^-1^), indicating the stronger Si–C bond. The stretching vibrations of the W–O bond appeared at 823 cm^-1^ and the bands at 570–650 cm^-1^ are related to W–O–W and O–W–O asymmetric stretching vibrations of the WO_4_^2-^ anion^48^.

The Si–C stretching vibration was observed at 816 cm^-1^ in g-SiC/Bi_2_WO_6_ catalyst which is slightly shifted to a higher frequency compared to g-SiC which indicates the immobilization of Bi_2_WO_6_ on the surface of g-SiC. The absorption bands that appeared at 582 and 470 cm^-1^ are related to Bi–O vibrations, and the absorption bands in the region of 729 cm^-1^ is corresponds to W–O vibration^49^.

The absorption band of Si–C appeared at 837 cm^-1^ in g-SiC/Na_2_WO_4_ catalyst. The W–O vibration of WO_4_^–2^ anion is observed in the 810 cm^-1^ and the band at 472 cm^-1^ is related to W–O–W vibration^50^.

**Figure 2:**
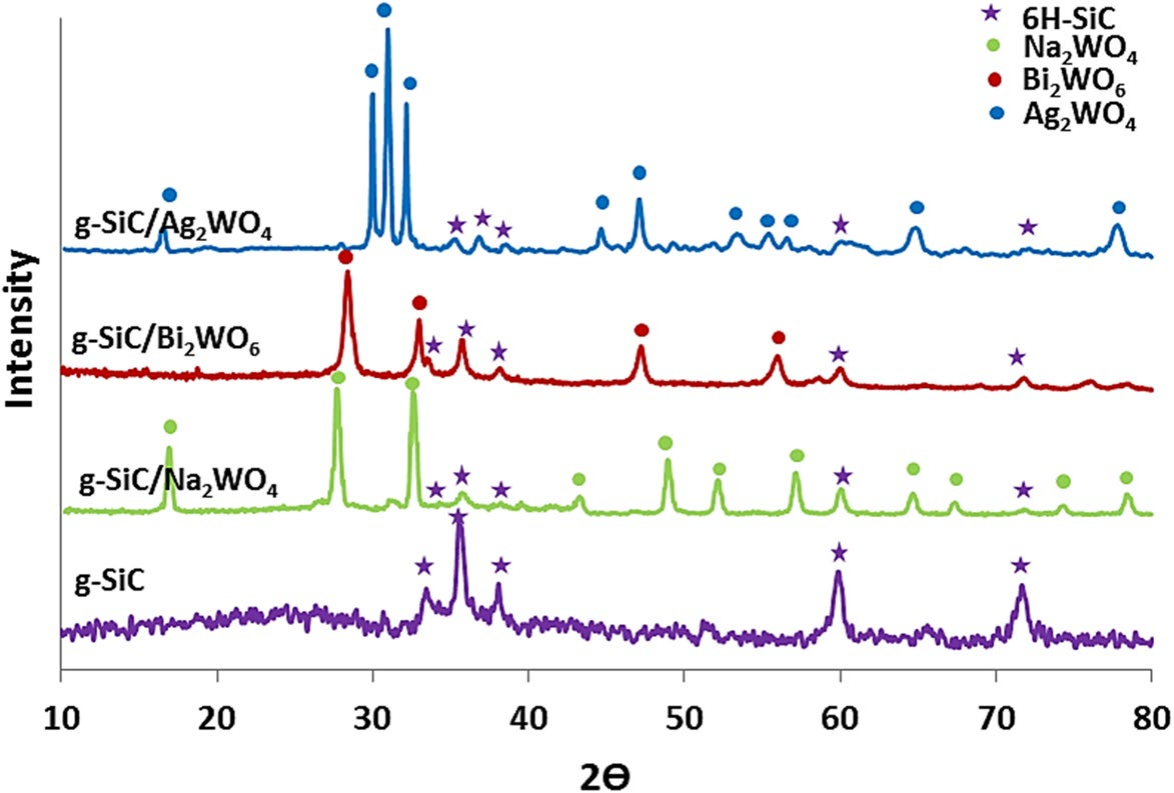
XRD diffraction patterns of g-SiC, g-SiC/Ag_2_WO_4,_ g-SiC/Bi_2_WO_6_, and g-SiC/Na_2_WO_4_.

Figure 2 shows the XRD diffraction pattern of g-SiC/Ag_2_WO_4,_ g-SiC/Bi_2_WO_6_, and g-SiC/Na_2_WO_4_ compared to g-SiC. The Ag_2_WO_4_ diffraction peaks were observed at 2Ɵ of 16.65, 29.95, 30.95, 33.15, 44.55, 46.15, 53.7, 55.6, 56.75, 65.01, and 77.9° in g-SiC/Ag_2_WO_4_ which represent the mixture of α and β phases of Ag_2_WO_4_ (JCPDS #34-0061; orthorhombic α- Ag_2_WO_4_) and (JCPDS #33-1195; hexagonal β- Ag_2_WO_4_)^19,48^.

In the g-SiC/Bi_2_WO_6_ sample, the diffraction peaks observed at 2Ɵ of 28.35, 32.95, 47.20, 56.16, 58.77, 76.06, and 78.61° indicate the (131), (200), (202), (310), (226), (113), and (139) planes of the orthorhombic structure of Bi_2_WO_6_ (JCPDS #39-0256)^49^.

Figure 2 shows that in g-SiC/Na_2_WO_4_ sample, the diffraction peaks of sodium tungstate were observed at 16.95, 27.75, 32.65, 43.40, 48.95, 52.05, 57.05, 59.95, 64.75, 67.45, 74.35, and 78.30° which indicate the (111), (220), (311), (331), (422), (511), (440), (620), (533), (444), and (642) planes (JCPDS# 12-772)^51^.

In all samples, the weak diffraction peaks of the hexagonal 6H-SiC structure were observed which indicates the covering of g-SiC surface with tungstate particles. 6H-SiC is a hexagonal polytype of SiC with stacking sequence repeats every six bilayers along the c-axis direction. The diffraction peaks of the g-SiC were observed at 31.61, 35.58, 37.98, 60.08, and 71.76 representing the (101), (102), (103), (110), and (202) planes in the hexagonal structure of g-SiC (JCPDS#-29-1128)^42–47^. The high amounts of immobilized tungstates (AWO) on the surface of g-SiC have led to the observation of weak diffractions from 6H-SiC in these composites.

The lattice fringe data were recorded in Table S1.

**Figure 3:**
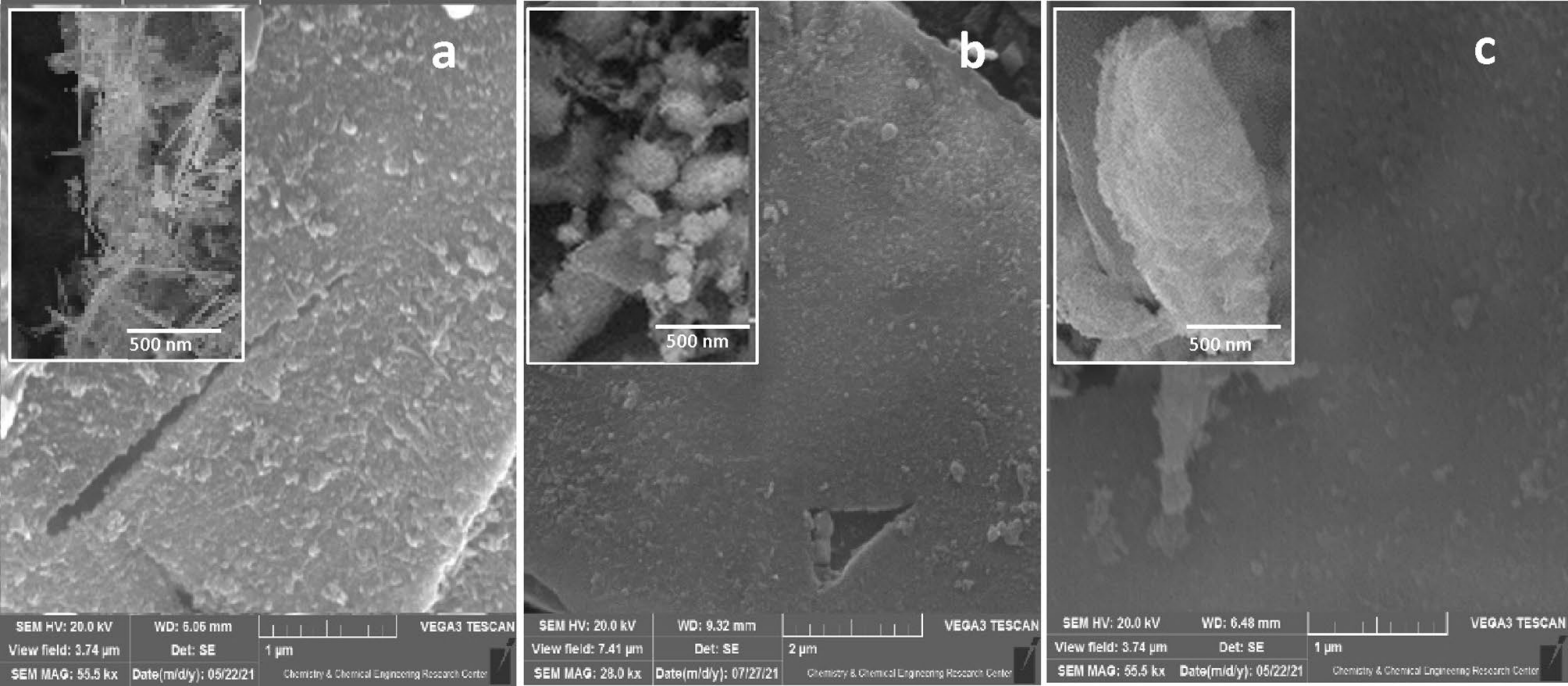
SEM images of g-SiC/Ag_2_WO_4_ (**a**)_,_ g-SiC/Bi_2_WO_6_ (**b**), and g-SiC/Na_2_WO_4_ (**c**).

Figure 3 shows the SEM images of synthesized catalysts. As shown in Fig. 3, needle-like, nanoparticle, and plate-like morphologies of Ag_2_WO_4,_ Bi_2_WO_6_, and Na_2_WO_4_ were observed on the surface of g-SiC in g-SiC/Ag_2_WO_4,_ g-SiC/Bi_2_WO_6_, and g-SiC/Na_2_WO_4_ catalysts, respectively.

**Figure 4:**
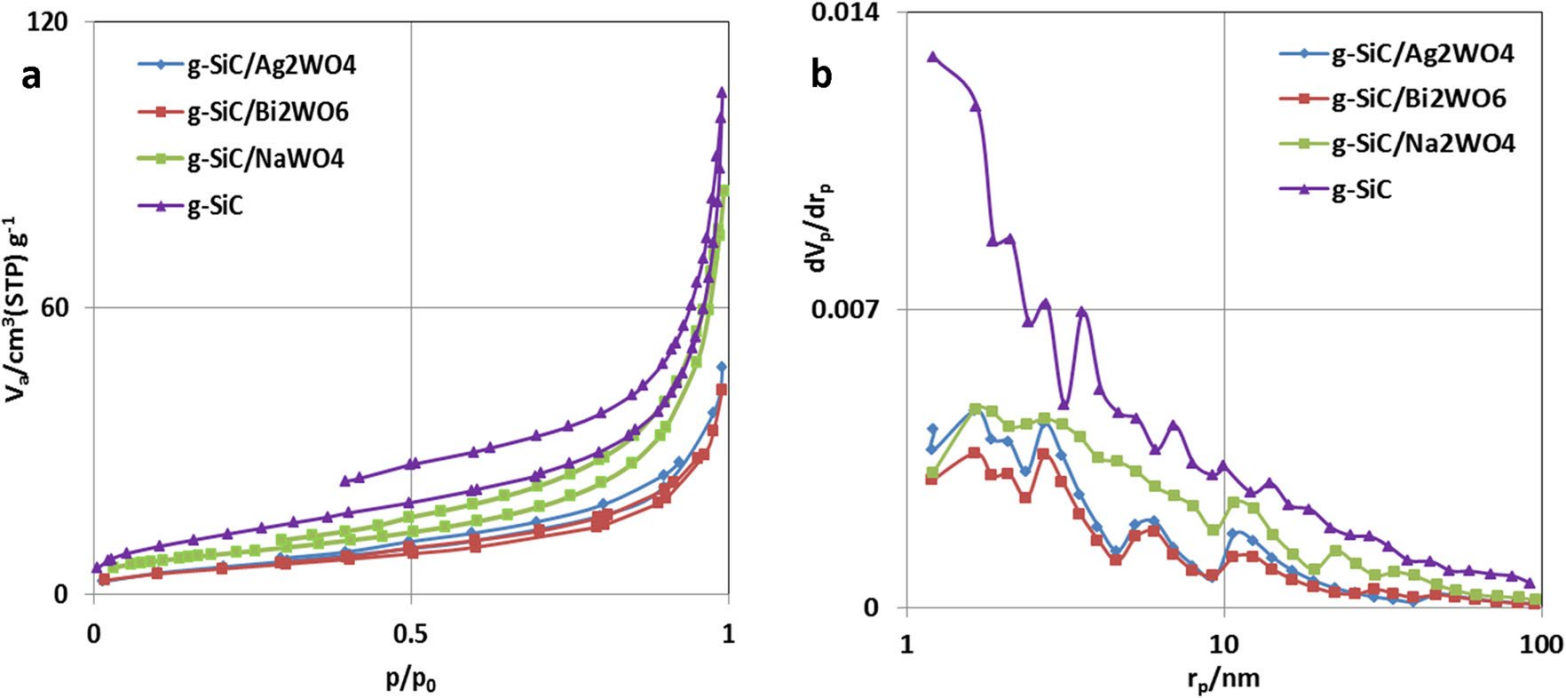
N_2_ adsorption/desorption isotherms and pore size distribution curves of g-SiC, g-SiC/Ag_2_WO_4,_ g-SiC/Bi_2_WO_6_, and g-SiC/Na_2_WO_4_.

Figure 4a displays the N_2_ adsorption/desorption isotherms of g-SiC, g-SiC/Ag_2_WO_4,_ g-SiC/Bi_2_WO_6_, and g-SiC/Na_2_WO_4_, which shows the decrease of surface area after immobilization of AWO on the surface of g-SiC. These results can be justified by the filling of pores in g-SiC by AWO particles, which is shown in pore size distribution curves (Fig. 4b). In g-SiC/Na_2_WO_4_, different pore distribution and higher surface area was observed, indicating the formation of new porosity of surface due to the larger size of Na_2_WO_4_ particles (Fig. 4b and Fig. 3c).

**Figure 5:**
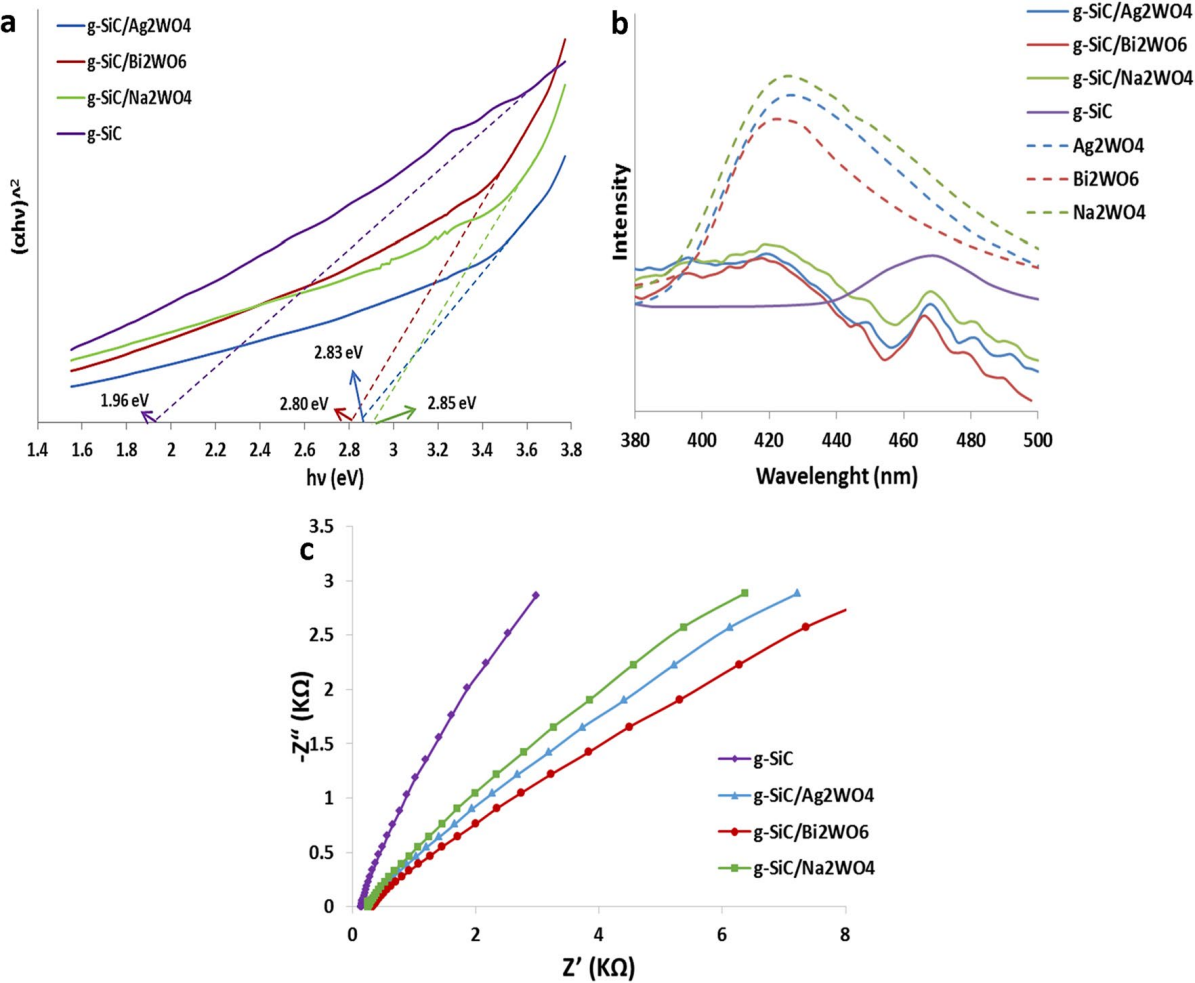
Band gap (**a**), photoluminescence (PL) spectra (**b**), and Nyquist plots (EIS) (**c**) of g-SiC, g-SiC/Ag_2_WO_4,_ g-SiC/Bi_2_WO_6_, and g-SiC/Na_2_WO_4_.

Figure 5a illustrates the band gaps of synthesized catalysts based on UV–Vis drift reflectance spectra. As can be seen, the band gaps of g-SiC/Ag_2_WO_4,_ g-SiC/Bi_2_WO_6_, and g-SiC/Na_2_WO_4_ are calculated as 2.83, 2.80, and 2.88 eV, respectively (Fig. 5). Results show the decreases of band gap of tungstate compounds after stabilization on the surface of g-SiC. The band gap of Ag_2_WO_4_, Bi_2_WO_6_, and Na_2_WO_4_ are reported 3.1, 2.89, and 3.7 eV, respectively^26,50,52^ which decrease after forming a heterojunction with g-SiC (band gap = 1.99 eV).

Photoluminescence (PL) spectra of synthesized samples were shown in Fig. 5b to evaluate the degree of charge recombination^53^. As shown in Fig. 5b, the PL intensities of g-SiC/AWO samples decrease significantly in comparison with the pure Ag_2_WO_4_, Bi_2_WO_6_, and Na_2_WO_4_, which indicates that the immobilization of tungstates on the g-SiC surface decrease the recombination rate of photogenerated electron-holes. In g-SiC/AWO samples, photogenerated electrons could be transferred efficiently from tungstates to g-SiC due to the high charge carrier of the g-SiC sheets.

The efficiency of photogenerated electron–hole separation of synthesized samples was further studied by the electrochemical impedance spectroscopy (EIS)^54^. As shown in Fig. 5c, the Nyquist plots of the g-SiC/AWO samples exhibit the smaller resistance than g-SiC, indicating the better separation efficiency of photogenerated charge carriers and therefore enhanced photocatalytic activities.

## Photocatalytic results

**Figure 6:**
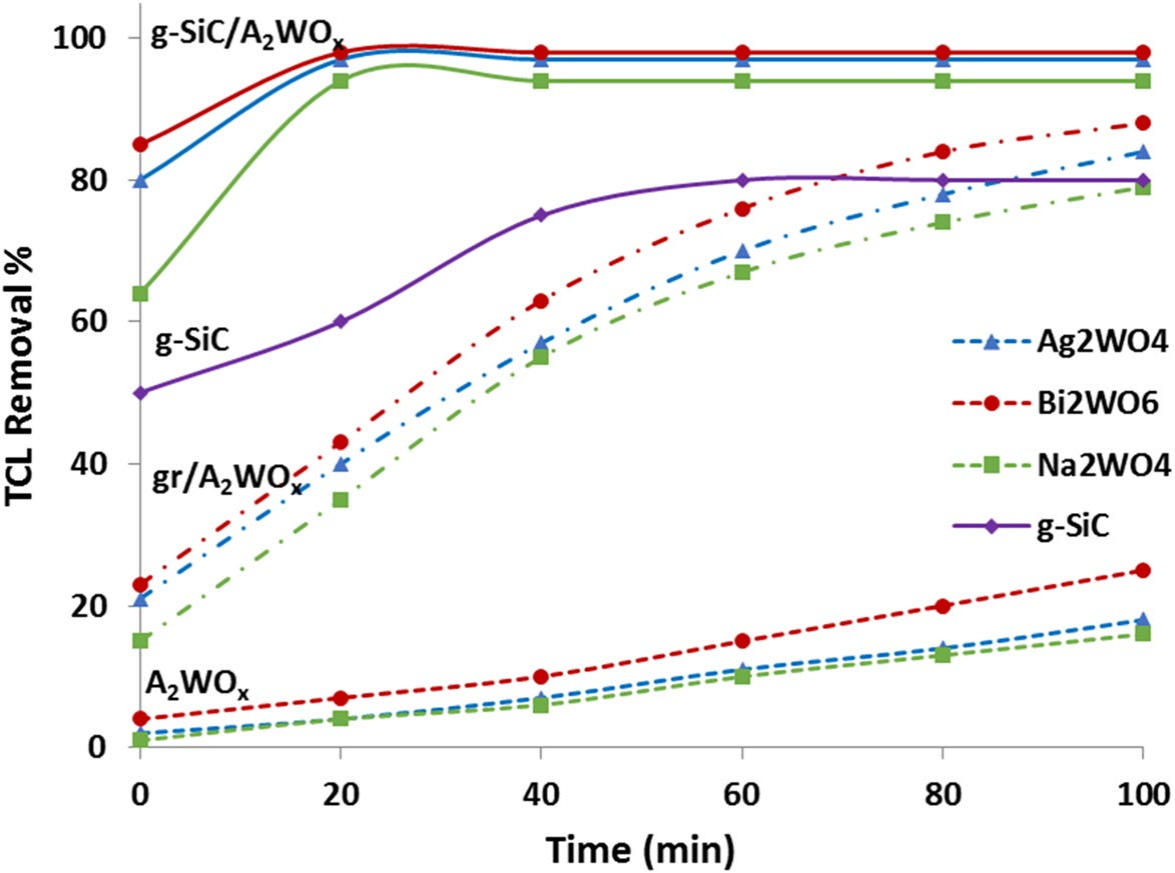
Comparison of photocatalytic performances of AWO, gr/AWO, g-SiC, and g-SiC/AWO composites in removal of 50 ppm TCL.

The photocatalytic performance of g-SiC, g-SiC/Ag_2_WO_4,_ g-SiC/Bi_2_WO_6_, and g-SiC/Na_2_WO_4_ in comparison with Ag_2_WO_4_, Bi_2_WO_6_, and Na_2_WO_4_ and their graphene composites (gr/AWO) were reported in Fig. 6. As can be seen, immobilization of tungstate catalysts on the surface of g-SiC enhances the photocatalytic degradation of TCL and 97, 98, and 94% removal of TCL (50 ppm) were observed for g-SiC/Ag_2_WO_4,_ g-SiC/Bi_2_WO_6_, and g-SiC/Na_2_WO_4_ after 20 min, respectively (Fig. 6). While, Ag_2_WO_4_, Bi_2_WO_6_, and Na_2_WO_4_ show the weak photocatalytic performances in the removal of high concentrations tetracycline (50 ppm), and 16, 25, and 18% TCL removal were observed after 100 min (Fig. 6). These results show the good effect of g-SiC as catalyst support. Figure 6 also shows that g-SiC as a metal-free catalyst represents better photocatalytic activity than tungstate catalysts and can remove 80% of TCL in a shorter time (30 min). This shows that g-SiC does not only play the role of catalyst support and acts as a photocatalyst to create a heterojunction with tungstates, which can improve the catalytic performances of tungstate photocatalysts (Fig. 6).

To better understand the effect of g-SiC on the photocatalytic performance of g-SiC/AWO composites, the photocatalytic properties of immobilized Ag_2_WO_4_, Bi_2_WO_6_, and Na_2_WO_4_ on the surface of graphene (gr/AWO) were also compared with g-SiC/AWO composites (Fig. 6). As we know, graphene along with other semiconductors like tungstates can improve the photocatalytic properties by increasing the transfer of electron and decreasing the rate of electron–hole recombination^24–28^. Therefore, better photocatalytic activities can be seen in gr/AWO composites than in tungstates alone (Fig. 6).

As seen in Fig. 6, replacing siligraphene (g-SiC) instead of graphene (gr) shows significant increases in photocatalytic properties. The g-SiC is a graphene structure in which half of its C atoms have been replaced by Si atoms. This graphene structure can increase the catalytic activity due to its high electron mobility. Besides charge transfer capability, also photogenerated charge lifetime contributes to the photocatalytic reaction. Interactions between g-SiC and Ag_2_WO_4,_ Bi_2_WO_6_, or Na_2_WO_4_ can reduce the rate of electron–hole recombination and improve photocatalytic performance. Also, π electrons in the siligraphene structure can form the π back-bonding with metal atoms and increase the electron–hole separation and enhance the photocatalytic activity^55^.

In addition to the advantages of graphene structure in g-SiC composites, the heterojunctions created with g-SiC reduce the band gap and can enhance the photocatalytic activity by shortening the electron transfer distance through the Z-scheme mechanism which will be explained later in this discussion.

Another mechanism that plays a role in enhancing the catalytic properties of g-SiC composites (g-SiC/AWO) is related to the surface charges in the g-SiC structure. According to our previous report, due to the electronegativity difference between Si and C in the g-SiC (siligraphene) structure, Si atoms have a partial positive charge and C atoms have a partial negative charge^7,10,42^. The dissolved O_2_ in a solution can adsorb on positive charged Si atoms in g-SiC, the O–O bonds dissociated and the oxygenated radicals were formed that can proceed the photocatalytic oxidation process^7,10,42^. As shown in Fig. 6, 64–85% TCL removal were observed at the time zero for g-SiC composites (g-SiC/AWO), indicating the degradation of TCL even at dark via oxidation of TCL by formed oxygenated radicals. While, in the graphene composites (gr/AWO), only 15–23% TCL removal was observed at dark due to the adsorption of TCL on the surface of graphene (Fig. 6).

As shown in Fig. 4, the BET results confirm the reduction of surface area in g-SiC/AWO composites. These data show that the increases in catalytic properties are related to the structural properties of immobilized tungstates on the surface of g-SiC, and the effect of the chemical structure was dominant over the effect of the surface area.

**Figure 7:**
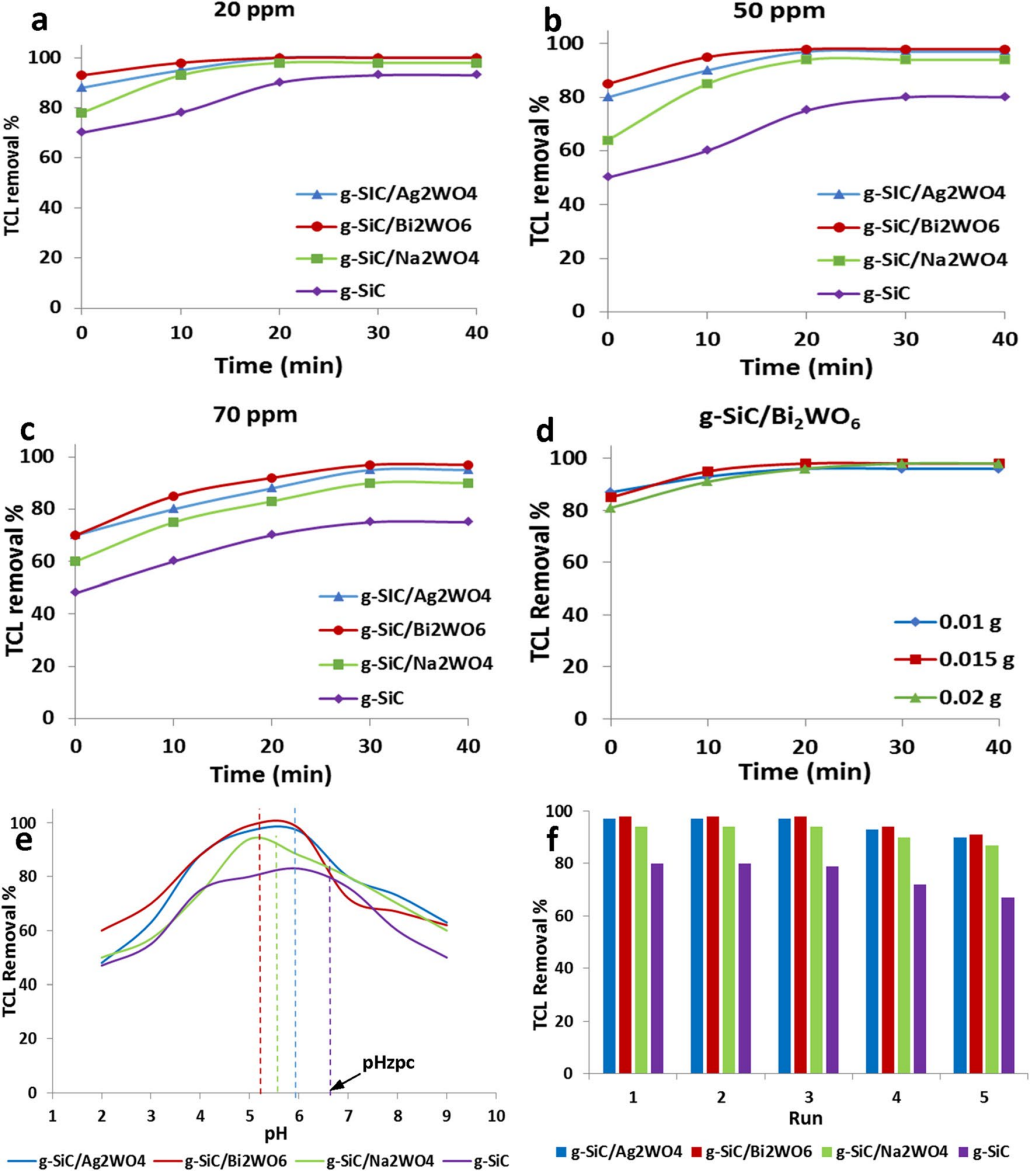
Photocatalytic removal of 20 ppm (**a**), 50 ppm (**b**), and 70 ppm (**c**) TCL in Visible light, different amounts of g-SiC/Bi_2_WO_6_ catalyst (**d**), effect of pH (**e**), and recovery of catalysts (**f**).

To achieve the best results, the reaction conditions were optimized in Fig. 7. Figure 7a–c represents the photocatalytic results in the removal of different concentrations of TCL. As can be seen, with the increase in TCL concentration, the removal has decreased. An increase in TCL concentration causes the saturation of the photocatalyst surface which decreases the photocatalytic activity.

The effect of catalyst amounts was also investigated by using g-SiC/Bi_2_WO_6_ as the best photocatalyst (Fig. 7d). Results show that increasing the amounts of photocatalyst from 0.01 to 0.015 g increases the catalytic performance. Increasing the amount of catalyst to 0.02 g only increases the rate of degradation (Fig. 7d).

The effect of pH in photocatalytic removal of TCL by g-SiC, g-SiC/Ag_2_WO_4_, g-SiC/Bi_2_WO_6_, and g-SiC/Na_2_WO_4_ catalysts were shown in Fig. 7e. As TCL has the several functional groups, different ionized species were formed at different pH. The H_2_TC^+^ species was observed at pH < 3.3, H_2_TC^0^ zwitterionic species was formed at pH 3.3–7.7, and HTC^−^/TC^2−^ species was produced at pH > 7.7^56,57^. As known, the surface charge of the catalyst becomes positive at a pH lower than pH_zpc,_ which can absorb the negative molecules. The pH_zpc_ of g-SiC, g-SiC/Ag_2_WO_4_, g-SiC/Bi_2_WO_6_, and g-SiC/Na_2_WO_4_ were measured 6.7, 5.9, 5.2, and 5.6, respectively. So, at pH_zpc_ of catalysts (pH = 5–7), H_2_TC^0^ as the main species of TCL can be adsorbed on the surface of catalysts via interaction of negative tricarbonyl amide groups of TCL with a positive charge of Si atoms on the g-SiC structure^10^. At a pH lower than 3.3, the TCL removal decreases due to the repulsion between the H_2_TC^+^ species of TCL and the positive surface of catalysts. Also, at a pH higher than 7.7, the photocatalytic performances were decreased which is related to electrostatic repulsion between the negative surface of catalysts and HTC^−^/TC^2−^ species of TCL (Fig. 7e).

To check the stability of the catalysts, their recovery was also investigated. As can be seen in Fig. 7f, no change in the photocatalytic efficiency can be observed after 3 runs. In the next runs, a slight drop in the photocatalytic activity is seen, which is related to the saturation of the catalyst surfaces. The g-SiC catalyst has a lower photocatalytic ability, so a greater drop in recovery is observed. A lower drop in the activity of tungstate catalysts (g-SiC/AWO) indicates the high activity of these photocatalysts in the continuous recovery.

**Figure 8:**
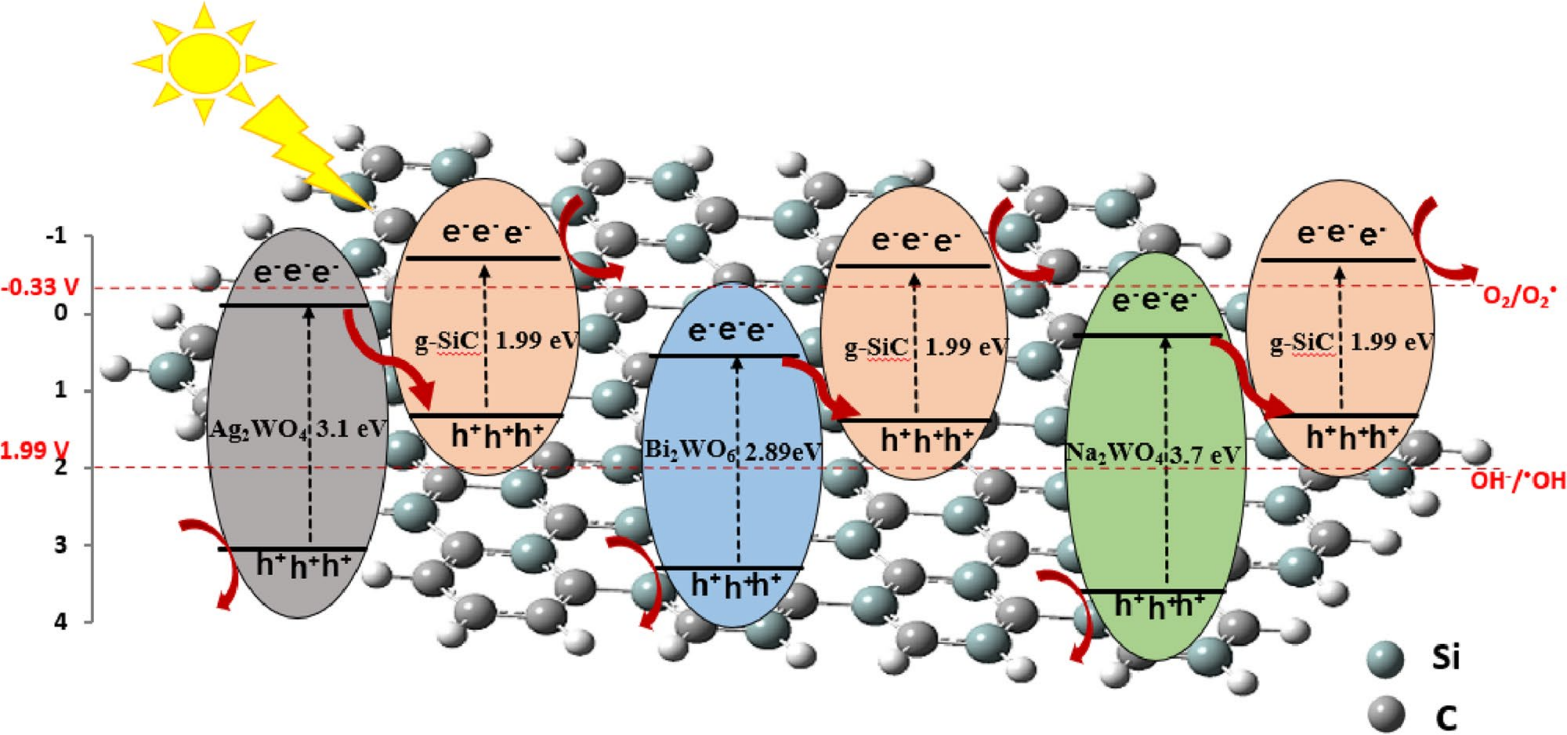
Z-Scheme mechanism of photocatalytic degradation by g-SiC/Ag_2_WO_4_, g-SiC/Bi_2_WO_6_, and g-SiC/Na_2_WO_4_ composites.

Figure 8 shows the proposed mechanism of photocatalytic degradation by g-SiC/Ag_2_WO_4_, g-SiC/Bi_2_WO_6_, and g-SiC/Na_2_WO_4_ composites. Based on band structures, these semiconductors are excited under visible light irradiation and electrons and holes are created in the valence (VB) and conduction (CB) bonds. The CB and VB potentials of g-SiC/AWO catalysts were predicted by the following equation:

$${\text{E}}_{{{\text{VB}}}} = X - {\text{ E}}_{{\text{e}}} + \, 0.{\text{5 E}}_{{\text{g}}}$$

$${\text{E}}_{{{\text{CB}}}} = {\text{ E}}_{{{\text{VB}}}} - {\text{ E}}_{{\text{g}}}$$

*X* is the electronegativity of the semiconductor and E_e_ is the energy of free electrons in the hydrogen scale (approximately 4.5 eV). The *X* value of g-SiC/Ag_2_WO_4_, g-SiC/Bi_2_WO_6_, and g-SiC/Na_2_WO_4_ are reported 6, 6.36, and 6.5 eV, respectively^48,50,58^. The E_g_ of Ag_2_WO_4_, Bi_2_WO_6_, and Na_2_WO_4_ were measured from UV–Vis spectra 3.1, 2.89, and 3.7 eV, respectively (Fig. 5b). As a result, E_CB_ and E_VB_ for Ag_2_WO_4_, Bi_2_WO_6_, and Na_2_WO_4_ have been calculated as − 0.05 and 3.05 eV, 0.41 and 3.30 eV, and 0.15 and 3.85 eV, respectively. The E_g_ of g-SiC was obtained at 1.99 eV from UV–Vis spectra (Fig. 5b) and E_VB_ was measured at 1.24 eV from XPS spectra (Fig. S1). As a result, the E_CB_ of g-SiC has been calculated as − 0.72 eV. Based on energy levels of g-SiC and its tungstate catalysts, the CB of g-SiC (− 0.72 V) is more negative than O_2_/·O_2_^−^ (− 0.33 V) and the VB of Ag_2_WO_4_ (3.05 V), Bi_2_WO_6_ (3.3 V), and Na_2_WO_4_ (3.85 V) are more positive than OH^-^/·OH (+ 1.99 V), so, the photogenerated e^−^ can reduce the O_2_ to form ·O_2_^−^ radicals and photogenerated h^+^ can oxidize OH^-^ to produce ·OH radicals. Results confirm the electrons in the CB of AWO combine directly with the holes in the VB of g-SiC, indicating the direct Z-scheme mechanism in the charge transfer path of photogenerated electron–hole in g-SiC/AWO heterojunctions.

Traditional BWO-based heterojunction is commonly performed via the Type-II strategy to accelerate the rate of charge transfer. These conventional heterojunction has a drawback that charge migration causes the photogenerated electron–hole to have weak redox ability^59–61^. In this novel Z-scheme heterojunction not only gives photocatalyst with superior redox ability, but also accelerates the migration of photogenerated electron-holes^62^. Figure 8 clearly shows that the heterojunctions created with g-SiC reduce the band gaps and can enhance the photocatalytic properties by shortening the electron transfer distance through the Z-scheme mechanism. The smaller band gap and shorter electron transfer path which enhances the stability of carrier transfer in Bi_2_WO_6_ is the reason for the better photocatalytic activity of this catalyst.

**Figure 9:**
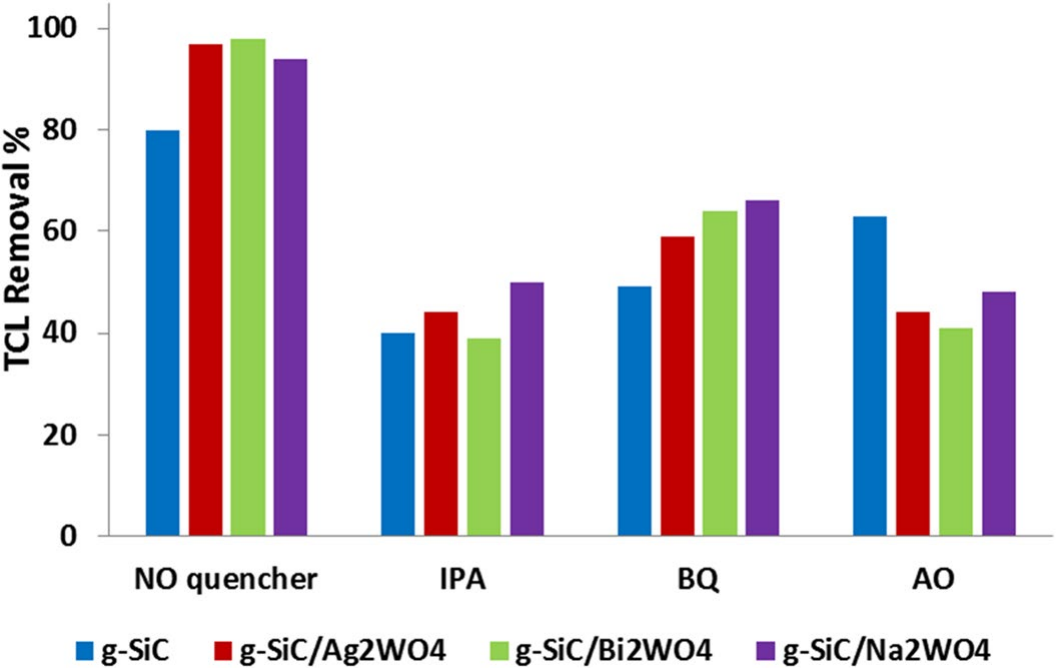
Quenching experiments of photocatalytic removal of TCL (ppm 50).

The quenching tests were also done to identify the active radicals involved in photocatalytic reaction for g-SiC/AWO catalysts (Fig. 9). The quenching results of g-SiC indicate the involvement of ^·^OH and ^·^O_2_^−^ radicals as the main species in the degradation of TCL. While, in g-SiC/AWO photocatalysts, ^·^OH and h^+^ are the reactive species in the photocatalytic reaction, and ^·^O_2_^−^ radical shows the lower involvement in the degradation of TCL (Fig. 9).

In general, it can be concluded that by using the g-SiC as catalyst support, new Z-scheme heterojunctions of tungstates (g-SiC/AWO) can be developed to find out the visible light degradation of high concentration of antibiotics by improving the charge separation, enhancing the electron transfer in Z-scheme heterojunction, and decreasing the rate of photogenerated electron–hole recombination.

## Methods

### Materials and instruments

Perlite (SiO_2_: 98%, porosity: 85%) was used as natural precursor of Si source and citric acid as a C source were purchased from Merck. Ethanol (96%), Mg powder (0.3–0.06 μm), hydrochloric acid (37%), and hydrofluoric acid (40%) were obtained from Merck.

FT-IR spectra were obtained from Bruker, Vector instrument. Scanning electron microscopy (SEM) was used to study the morphology of samples by TESCAN, VEGA3 microscope armed with the X-ray energy dispersive spectroscope (EDX), and TEM microscopy was done using Zeiss, EM10C microscope. Crystalline phases were identified by BrukerAxs, D8 Advance X-ray diffractometer (XRD) using CuK_α_ radiation. Raman spectra were obtained with Bruker, Senterra Raman spectrometer with a 785 nm laser. N_2_ adsorption/desorption isotherms and the pore size distributions were recorded by Belsorp mini instrument. XPS analysis was recorded from ESCALAB 250Xi Thermo Scientific system (MgKα = 1253.6 eV).

### Synthesis of g-SiC (siligraphene)

Citric acid was used as a carbon source and prepared by dissolving 0.8 g of citric acid in 100 mL of distilled water. Perlite powder was first washed with HCl (1 M) solution to remove its impurities and dried at 70 °C for 8 h. Then, 2 g of perlite was added to 200 mL of citric acid (8 g/L) solution and sonicated for 15 min. The powder filtered and dried at oven. To better penetration of citric acid to perlite pores, this process repeated 5 times. Finally, the mixture was dried at 80 °C for 24 h. The obtained powder was put in a furnace at 800 °C (5 °C /min) for 1 h under an N_2_ atmosphere to obtain the SiO_2_/carbon composite. To form the Si–C bond, the composite was mixed with Mg powder with the Si:Mg ratio of 1:2 and heated at 800 °C for 5.5 h under an N_2_ atmosphere (5 °C /min). To eliminate the impurity of MgO, the produced g-SiC was immersed in HCl solution (2 M) for 48 h and then washed with distilled water to remove MgCl_2_. The residual silica was removed by immersing the produced g-SiC in 2 M HF solution. Afterward, the g-SiC (siligraphene) was filtered and dried in a vacuum oven at 60 °C for 24 h.

### Synthesis of g-SiC/AWO (A=Ag, Bi, Na)

For stabilization of Ag_2_WO_4_ and Bi_2_WO_6_ on g-SiC (siligraphene), 0.2 g of synthesized g-SiC was sonicated for 10 min in a certain amount of distilled water. Silver or bismuth solution was prepared by dissolving 0.1 g of silver or bismuth nitrate in 25 mL of distilled water and stirring for 0.5 h. Then, Ag or Bi solution was added to g-SiC suspension and stirred for 0.5 h. Tungsten solution was also prepared by dissolving 0.1 g of sodium tungstate in 25 mL of distilled water for 0.5 h, then added dropwise to Ag/g-SiC or Bi/g-SiC mixture under sonication. The final mixtures were placed in an oven at 160 °C for 5 h. The solid powder was filtered and dried at 70 °C.

The g-SiC/Na_2_WO_4_ was also synthesized by adding 0.1 g of sodium tungstate to g-SiC suspension under sonication. The mixture was heated at 160 °C for 5 h. The solid product was filtered and dried at 70 °C.

### Photocatalytic experiments

To investigate the photocatalytic performance, the removal of tetracycline (TCL) as a drug pollutant were evaluated. In a batch experiment, a certain amount of g-SiC/Ag_2_WO_4_, g-SiC/Bi_2_WO_6_, and g-SiC/Na_2_WO_4_ photocatalysts was dispersed in 10 mL of tetracycline (TCL) solution under stirring. The solution saturates with oxygen by air bubbling into the system and irradiated by visible light. To optimize the photocatalytic reaction, different concentrations of TCL (20, 50, and 70 ppm) were tested by using 0.01, 0.015, and 2 g of catalyst at pH of 2–12. Before irradiation, all samples were stirred at dark for 5 min, and then the photocatalytic process was performed. The concentration of dye in the solution was measured by UV–Vis spectroscopy after each 20 min. After each 10 min, the concentration of TCL was measured by UV–Vis spectroscopy at 357 nm. The TCL removal percent was determined using the following equation:

$${\text{Removal \% }} = \left[ {\frac{{\left( {C_{0} - C_{t} } \right)}}{{C_{0} }}} \right]*100\%$$

where *C*_*0*_ and *C*_*t*_ are the initial and equilibrium TCL concentrations.

Quenching experiments were done by using 2 Mm of isopropanol (IPA) as hydroxyl radical (^·^OH) scavenger, 1,4-benzoquinone (BQ) as superoxide radical anions (^·^O_2_^−^) scavenger, and ammonium oxalate (AO) as the scavenger of the hole (h^+^).

## Conclusion

The new Z-scheme heterojunctions of siligraphene (g-SiC) with different tungstates (Ag_2_WO_4_, Bi_2_WO_6_, and Na_2_WO_4_) were synthesized and their photocatalytic properties were investigated in the removal of tetracycline. Based on the photocatalytic degradation results, the photocatalytic potentials of tungstates catalysts were promoted by immobilizing on the surface of g-SiC. The graphenic structure of g-SiC can improve photocatalytic performance by increasing the electron transfer and decreasing the rate of electron–hole recombination. Also, the π back-bonding of g-SiC with metal atoms increases the electron–hole separation to enhance the photocatalytic activity. Furthermore, the heterojunctions created with g-SiC reduce the band gap and can enhance the photocatalytic activity by shortening the electron transfer distance through the Z-scheme mechanism. The optimized g-SiC/AWO composites exhibited high photocatalytic performances and 97, 98, and 94% of high concentrations tetracycline (50 ppm) were removed after 20 min by using only 10 mg of g-SiC/Ag_2_WO_4_, g-SiC/Bi_2_WO_6_, and g-SiC/Na_2_WO_4_ catalysts, respectively. The g-SiC/Bi_2_WO_6_ photocatalyst shows the highest photocatalytic activity due to the smaller band gap and shorter electron transfer path.

## Supplementary Information

Supplementary Information.

## Data Availability

All data generated or analyzed during this study are included in this published article.
