# Supplementary material for: Novel synthesis of siligraphene/tungstates (g-SiC/AWO) with promoted transportation of photogenerated charge carriers via direct Z-scheme heterojunctions
Source: Sci Rep. 2023 Jun 20;13:10022. doi: 10.1038/s41598-023-37170-5 (PMC10282037; doi:10.1038/s41598-023-37170-5)
Supplement: Supplementary file 1 — Supplementary Information. [file 41598_2023_37170_MOESM1_ESM.pdf]

Novel synthesis of siligraphene/tungstates (g-SiC/AWO) with promoted transportation of photogenerated charge carriers via direct Z-scheme heterojunctions

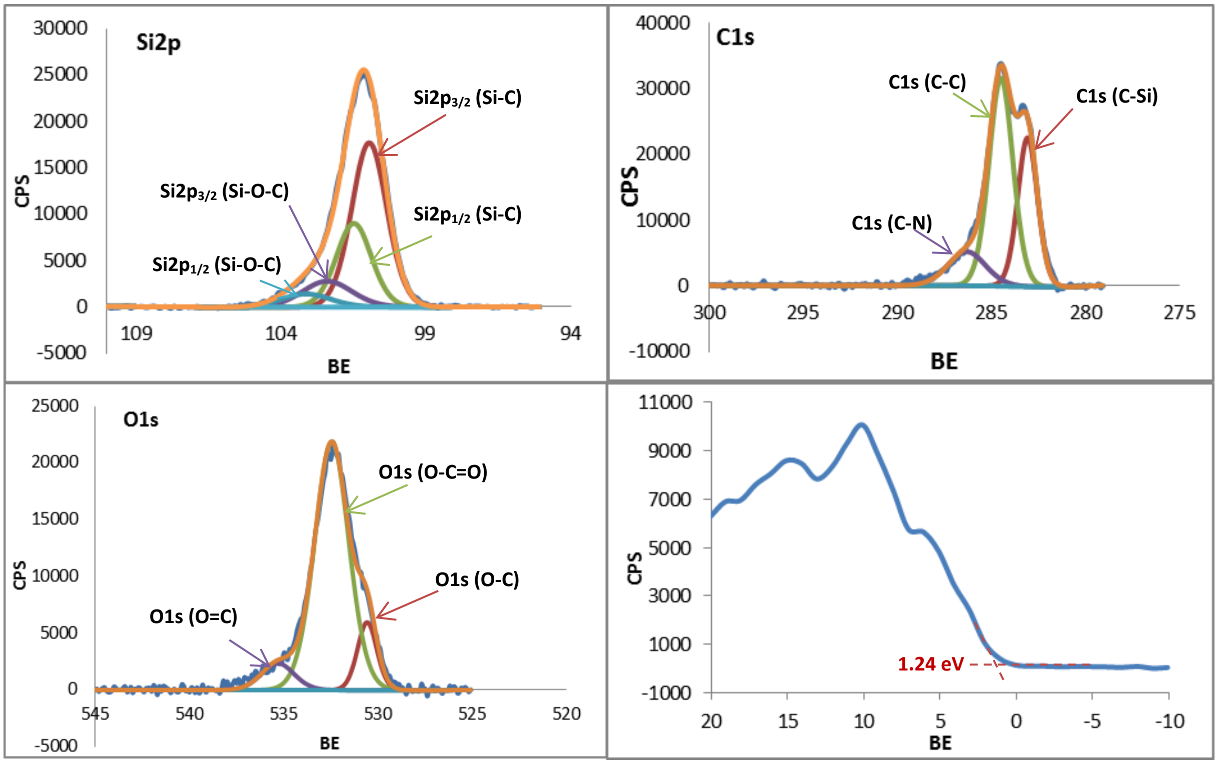

Fig. S1 XPS spectra of synthesized g-SiC

Table S1 Lattice fringe data of synthesized g-SiC/A_2WO_x composites.

Sample	phase	JCDPS No.	2Ѳ	h	k	l
**g-SiC**	Hexagonal 6H-SiC	29-1128	31.61	1	0	1
			35.58	1	0	2
			37.98	1	0	3
			60.08	1	1	0
			71.76	2	0	2
**g-SiC/Ag_2WO_4**	orthorhombic α- Ag_2WO_4	34-0061	29.95	0	0	2
			30.95	2	3	1
			33.15	4	0	0
			46.15	4	0	2
			53.7	3	2	3
			55.6	2	0	6
	hexagonal β- Ag_2WO_4	33-1195	16.65	0	1	1
			29.95	0	2	2
			30.95	2	2	0
			44.55	0	4	2
			56.75	2	4	2
			65.01	2	2	4
**g-SiC/Bi_2WO_6**	Orthorhombic Bi_2WO_6	39-0256	28.35	1	3	1
			32.95	2	0	0
			47.20	2	0	2
			56.16	3	1	0
			58.77	2	2	6
			76.06	1	1	3
			78.61	1	1	9
**g-SiC/Na_2WO_4**	Na_2WO_4	12-772	16.95	1	1	1
			27.75	2	2	0
			32.65	3	1	1
			43.40	3	3	1
			48.95	4	2	2
			52.05	5	1	1
			57.05	4	4	0
			59.95	6	2	0
			64.75	5	3	3
			67.45	4	4	4
			74.35	6	4	2
			78.30			
